# A study of reasons why teenagers living in the countryside become alcoholics at an early age

**DOI:** 10.1192/j.eurpsy.2022.1099

**Published:** 2022-09-01

**Authors:** L. Baranskaya, Y. Babyshkina

**Affiliations:** 1 Ural State Medical University, Psychiatry, Psychotherapy And Narcology, Yekaterinburg, Russian Federation; 2 Ural State Medical University, Psychiatry, Psychotherapy Fnd Narcology, Yekaterinburg, Russian Federation

## Abstract

**Introduction:**

Irreversible damage is caused to the physical and psychic health of teenagers who become alcoholics at an early age. They later become addicted to alcohol which factor leads to the risk of development of chronic diseases, medical and social consequences

**Objectives:**

Monitoring of subjective reasons and widespread frequency of drinking alcohol by teenagers living in the countryside aged 13-17

**Methods:**

One thousand sixty two teenagers volunteered to take part in the study: 55.0% female and 45.0% male. Anonymous survey was used. The forms made up by the Center of Monitoring of Detrimental Habits among Children and Teenagers (Moscow, Russia)

**Results:**

The results of the study undertaken made it possible to see the main reasons for the early formation of drinking habits and the age at which teenagers start to drink. The main reason is the socially widespread myth that alcoholic drinks of a high quality are not detrimental to health at all. One third of the teenage respondents think so. The 42.3% of the young men and 65.3% of the girls show that first drank alcoholic drinks were in the family. Teenagers living in the countryside start to drink at the age of 13 to 17 including. The peak for females is 15-16, for males it is 13-16

**Conclusions:**

Taking into account the significant role of the family in the formation of early drinking habits of teenage males and females living in the countryside and the stable attitudes in family traditions would not help form in teenagers a motivated refusal to drink alcohol

**Disclosure:**

No significant relationships.

